# Factors affecting professional ethics in nursing practice in Iran: a qualitative study

**DOI:** 10.1186/s12910-015-0048-2

**Published:** 2015-09-09

**Authors:** Ali Dehghani, Leili Mosalanejad, Nahid Dehghan-Nayeri

**Affiliations:** 1 Nursing Department, Nursing and Paramedical school, Jahrom University of Medical Sciences, Jahrom, Iran; 2 Mental Health Department, Nursing and Paramedical school, Jahrom University of Medical Sciences, Jahrom, Iran; 3 Nursing Management Department, School of Nursing and Midwifery, Tehran University of Medical Sciences, Tehran, Iran

## Abstract

**Background:**

Professional ethics refers to the use of logical and consistent communication, knowledge, clinical skills, emotions and values in nursing practice. This study aimed to explore and describe factors that affect professional ethics in nursing practice in Iran.

**Methods:**

This qualitative study was conducted using conventional content analysis approach. Thirty nurses with at least 5 years of experience participated in the study; they were selected using purposive sampling. Data were collected through semi-structured interviews and analyzed using thematic analysis.

**Results:**

After encoding and classifying the data, five major categories were identified: individual character and responsibility, communication challenges, organizational preconditions, support systems, educational and cultural development.

**Conclusions:**

Awareness of professional ethics and its contributing factors could help nurses and healthcare professionals provide better services for patients. At the same time, such understanding would be valuable for educational administrators for effective planning and management.

## Background

Nursing mission is to provide high quality healthcare and maintaining and improving community health [1]. Ethics is considered as an essential element of all healthcare professions including nursing. Thus, it has a central role in nurses’ moral behavior toward patients, which strongly influences on patients’ health improvement [2]. Professional ethics constitutes legitimate norms or standards that govern professional behavior of both client and non-client [3]. Indeed, professional ethics addresses obligations of a profession towards people who are served [4].

An inherent part of nursing is to respect human values, rights and dignity [5]. From a clinical point of view, nursing has three basic principles of caring, namely ethics, clinical judgment, and care [6]. Vinson [7] points to five elements that are epistemological and fundamental to nursing, which include the following: knowledge of nursing, art of nursing, individual knowledge, ethics of nursing, and sociopolitical knowledge. From moral and philosophical perspective, nursing ethics incorporates using of critical thinking and logical reasoning in clinical practice on the basis of values [7].

Nursing ethics might also be considered as competency in nurses without any direct impact on their clinical activities, which could be separated from practical duties of nursing. However, such ethics are highly interwoven with clinical practices that cannot be alienated from them [8]. Lemonidou *et al.* [9] suggest that ethical commitment to care is an integral part of nursing practice in nurse-patient relationship.

Nowadays, health care settings are changing rapidly. Thus, nurses are facing ethical challenges in healthcare that put them at risk of ethical conflict [10]. Although meeting the requirements of professional ethics in patients’ care is essential, studies revealed that standards of professional ethics are not observed in nursing practices. Indeed, standards and criteria of professional ethics are not considered based on patients’ preferences and culture [11]. According to previously conducted studies, nurses had poor attachment to professional ethics. Sokhanvar [12] reported that nursing awareness and application of ethical principles in patient’s care and clinical decisions were not desirable in Fars, Iran. Additionally, nurses were not interested in applying ethical knowledge in their work [12]. Tefagh et al. [13] found that safe medication administration by Iranian nurses was significantly poor and lacked adherence to the professional ethics. A comparative study on nurses’ perceptions of ethical problems in China and Switzerland revealed that there were differences in some ethical concepts including culture and faith. Chinese nurses were more nervous, sad and dissatisfied during and after the work compared to nurses from Switzerland. However, both groups experienced ethical problems of poor communication with patients due to heavy workload [14, 15]. Another study reported that nurses might confront with various problems during their works [15]. Thus, ethical issues should be taken seriously as a basic requirement. On the other hand, the most comprehensive and complete approach to observe ethical standards is qualitative approach in which participants share their experiences [16, 17]. Such information helps administrators promote professional ethics. This study aimed to explore and describe factors affecting professional ethics in nursing practice in Iran.

## Methods

This qualitative study was conducted using conventional approach of content analysis. It has been intended to explore and describe factors affecting professional ethics in clinical practice. In general, content analysis is used when the objective of a study is to describe a phenomenon, and there are limited ideas [18] or fragmented knowledge about it [19]. Additionally, the phenomenon of professional ethics for nursing and affecting factors has vague aspects, which should be clarified through content analysis.

Participating nurses were selected by purposive sampling from hospitals affiliated to Jahrom University of Medical Sciences in Jahrom, Fars, Iran. A total of 30 nurses including 25 female and five male nurses with at least 5 years of experience participated in the study. The sample size was chosen based on the data saturation. Data were collected using individual face to face and semi-structured in-depth interviews. Each interview took between 60 and 100 min. All interviews were conducted in the participant’s workplace in a quiet setting. Firstly, interviews were started with main questions in accordance with participants’ statements. Then, it was continued by probing questions. All interviews were initiated with this question: “as a nurse please tells me about the ethical issues you have faced in your workplace”. As the interview progress, these questions were asked: what factors affect professional ethics in your clinical care? All interviews were recorded and transcribed immediately.

Conventional approach for data analysis was implemented; no structure was used for categorizing data. This approach was carried out over three phases including: preparation, organizing and writing the report. In the preparation phase, each interview was treated as a unit of analysis. The recorded interviews were transcribed precisely and read several times to gain general impression. In the organizing phase, unites of meaning for each interview was highlighted, condensed, and openly coded. Then, codes with similar meanings were arranged into subcategories and main categories. Finally, the latent meaning of the data was reported in the reporting phase [19].

Conformability of findings was evaluated to achieve the reliability of collected data [20]. To achieve credibility of findings, content analysis, selecting appropriate units of meanings, way of categorizing data, and making judgment about similarities and differences of categories are very important [21]. Accordingly, the credibility of findings of this study was evaluated through spending enough time for data collection and analysis. Member check was also performed; data analysis was carried out by the second author for peer check.

The approval of study was obtained from the Ethics Committee of Jahrom University of Medical Science. The participants were asked to sign a consent form; they were assured that they can withdraw from the study at any time.

## Results

**Fig. 1:**
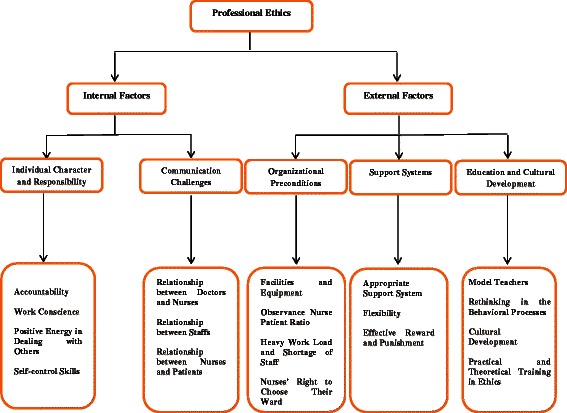
Factors affecting professional ethics in nursing

The findings highlighted two main themes: internal factors that deal with individual characters, responsibility and communication challenges; external factors that were reflected on organizational preconditions, support systems, educational and cultural development (Fig. 1).

### Internal factors: individual character and responsibility

In this category, were extracted accountability, work conscience, positive energy associated with others, and self-control skills in conflicting situations. Most nurses pointed to professional ethics and accountability as important features that contributed to making a background for the ethical context of healthcare setting.

Accountability: one of the participants with 11 years of experience mentioned that if nurses devoted themselves to their work sincerely, accepted their responsibility, and acted accordingly, the patients would receive medical care appropriately. For example, a nurse might use his/her hand (i.e. by putting it on the patient forehead) to see if the body temperature was normal instead of using a thermometer. Such inappropriate methods could jeopardize patient’s health, because there was high possibility of making mistake. Another participant with 10 years of experience about work conscience said: “I think that work conscience is a personal issue, and no one can be forced to accept it.” She continued “there are specific times when my shift at work is over, but I am still in the middle of the work. I take the responsibility of patients care and continue until I finish my duty, even if I have to stay more.” However, some nurses explain their shift is over and they have to wait for the next shift. In the interim, work conscience is another factor that is important in work discipline and generates sense of duty in the individual.

By character, we mean a set of behavior and manners of thinking that individuals use in their everyday life situations. Character is also determined by other characteristics that are unique to that individual; it has been established in him/her and is predictable. Regarding the character of an individual, the participants believed that personality is formed during childhood in both family and society. However, they added that the environment is responsible for changing 40–50 % after the formative years. Manager can adapt to the environment; for example, with a little encouragement can be a very good influence.

Regarding the possession of positive energy, a nurse said: “in my opinion, a nurse should work in an environment in which marginal issues are excluded. In addition, it is brimmed with positive attitudes and energy. Because patients admitted to hospital are often suffering from disruption in health condition, and this is uncomfortable for them and their families. Therefore, nurses should provide care with positive energy for patients and their relatives, reinforce their spirit to recovery and create a sense of hope.

Regarding the internal control skills, a participant expressed: “A professional nurse has to be able to be considerate in different situations. For example, I had a patient suffering from multiple fractures. He was using bad words when he has severe pain. While I was doing his treatment, he even pushed me hard so that I felt down on the floor, but it did not make me upset. I did my best in spite of the patient’s behavior until I finally could reduce his pain.

### Internal factors: communication challenges

In this category, the following themes were extracted: communication between doctors and nurses, professional relationship among staff, nurse-patient relationship, and effective communication and interaction in the workplace.

In this regard, a participating nurse with 8 years of experience said: “a doctor found an error in a patient’s medical records. Although it was not my fault, he insulted me verbally.” she added: “Unfortunately, reporting such cases is not beneficial. Because authorities do not attend or respond to such instances in the healthcare system.”

Nurse- patient relationship: A participant with 12 years of experience stated: “I had an end stage patient. Even though the medical team was disappointed to him, and his level of consciousness was not at full stage, whenever I went to take care of the patient, I talked to him without receiving any response. The patients’ family told me that he opened his eyes when the nurse was giving medication and nursing care. I feel that the patient was waiting for someone who cared about him; the patient even in the absence of any communication could realize that someone had sympathy for him.”

Nurse-patient communication: A participant with 8 years of experience said: “I believe that good communication with patients has miraculous results. For example, I had a patient with cardiac and respiratory diseases. He had an excruciating pain that made him scream. While I was doing my job as a nurse, I talked to him in a soothing manner and kept telling that he would be alright. I thought, based on his behavior, talking to the patient was effective enough to somehow make him relaxed.”

### External factors: organizational preconditions

In this category, the following themes were chosen: facilities and equipment; observance of nurse-patient ratio; heavy work load and shortage of staff; nurses’ right to choose an appropriate ward.

Hospital facilities and equipment play an important role in establishing professional ethics. Non-standard equipment can interfere with providing proper care and may even mislead medical staff judgment about patients’ conditions. For example, we had a patient with kidney problems who had undergone surgery. While the patient was suffering from infection, temperature control device showed his fever lower than the actual degree. This seemingly simple incident could increase the length of hospitalization and hospital costs.

Inappropriate nurse-patient ratio and heavy work load: a participant with 11 years of experience stated that once we had 40 patients, while only 5 nurses were available for care. Suppose from the above number, only 25 patients were in need of special care every few minutes, how could such a limited number of nurses be able to respond to required demands of the patients? It was absolutely infuriating. To make the situation still worse, add 25 more people to the list of the patients, those who accompany patients to the hospital and frequently go to the stations at hospital wards and expect to receive proper answers for their questions whenever they wish.

Another participant with 14 years of nursing experience stressed on nurses’ rights to choose their own working places at hospitals. According to this person, this opportunity could affect the application of the professional ethics. He said: “I was supposed to work for 2 years as an obligatory practice after graduation. I worked in the emergency ward, in spite of my will.”

### External factors: support systems

In this category, the following themes were extracted: appropriate support system, flexibility and effective reward and punishment.

A participant with 12 years of experience said: “I believe that an effective support system should encourage us to observe the professional ethics. Whenever I face a problem, supervisors should support me. Also, a proper system of reward and punishment could help enhance experience of the professional ethics.”

Another participant highlighted flexibility as a necessity for nursing. He said: “nursing practice requires even if a patient is too much demanding or he has had challenges with us; we should never deprive him from our services.

Another aspect of supportive system, according to a participant with 15 years of experience was an efficient way of punishment and reward system. He said “It would be helpful for nurses to get a positive or negative feedback based on their professional behavior. If the monitoring system rewarded me when I did my duties efficiently, I would be encouraged to work 10 times more than I supposed to; otherwise, I lose my motivation. Just try it for six months and see the results”.

### External factors: educational and cultural development

In this category, these themes were extracted: model educators and attention to practical ethics through modeling the environment, re-thinking about behavioral processes, cultural development focusing on ethics, and specialized practical and theoretical training courses in ethics.

Regarding educator modeling and its impact on the development of morality, a participant with 13 years of experience stated that nursing instructors should be aware of the effects of training methods on trainees. He added “our instructor once forced male students to empty patients’ urine bags and change the bed sheets in the presence of patients’ companions and cleaning staff of the hospital. Meanwhile, he put the nurse under more pressure by repeating his order again and again. Such behaviors make negative impacts on our views of the job as a nurse.”

For the issue of re-thinking about behavioral processes, a participant with 10 years of experience commented “I had a patient with ventricular fibrillation. As physicians were not available at that time, I started resuscitation. It was successfully performed, and the patient is still alive. As I reflect on my deeds, I found it very important.” The nurse continued, “In another instance, I found an error in a physician’s prescription and since I was sure about the exact medication dosage, I made the correction.”

Regarding cultural development and ethics, a participant with 16 years of experience stated: “I had a critically ill patient who were supposed to be transferred to another hospital. I stayed with him until 4 pm, after my shift was over at noon; I had lunch after arriving home.” Such devotions or commitment to a profession can be strengthened by means of cultural development.”

A participant emphasized on the importance of ethical courses for nurses. He said “training nurses in services expected from them is necessary. Every year, CPR training is repeated for us in accordance with new protocols to keep us updated.” However, another participant’s talk was focused more on educating nurses in professional ethics. He mentioned, “Such a course should be taught on location to make nursing students familiar with accepted patterns of morality in their interactions with patients.

## Discussion

Factors effecting professional ethics in nursing practice have been identified in this study. The first main category of the findings was focused on the individual character and responsibility. It was emphasized on developing a sense of responsibility in nurses as a significant factor that influences professional behavior. Also, nursing literature indicated that creating professional commitment should be regarded as a necessary quality for nursing practice; nurses should be accountable for their decisions and outcomes. Such characteristics lead to better observance of professional ethics by nurses [22]. Indeed, most nurses believed that individual character and responsibility play an important role in sensitivity to the professional ethics compliance and moral development. Abbaszadeh et al. [23] emphasized that students who desire to enter into nursing profession should be checked for metacognitive features (e. g. personality) and be coordinated with nursing profession.

The second category is communication challenges among health care members. The participants highlighted effective relationship as the element of professional ethics. The researchers also believe that effective nursing is highly related to developing proper relationships among members of the health care system. In the absence of such attitudes, patient care will be adversely affected.

This study also indicates that patient’s assessment is one of the important measures in establishing rapport between nurse and patient [24]. In recent years, it has been emphasized on professionalism in nursing. Thus, health care system requires nurses who are able to develop relationships with the multidisciplinary professionals as well as patients and their families [25]. According to Doran et al. [26], nurses do not work solely. In other words, they should try to expand connections with other health care teams in order to enhance patients’ quality care [26]. In addition, Weaver and Morse [27] stated that interpersonal relationship is a vital factor in ethical sensitivity, and ignoring it may decrease the sensitivity. Also, participating students in the study conducted by Borhani et al. [28] expressed poor interpersonal communication as one of the barriers in achieving professional ethics. Sadeghi and Ashktorab [29] reported that poor communication between doctors and nurses and patients is a main part of the most raised ethical problems, which could lead to the violation of patients’ rights.

Organizational preconditions are the third category affecting professional ethics. Adib Haghbaghery et al. [30] stated that organizational structure should be compatible with nursing professional knowledge. When there are inappropriate organizational structures in health care systems, nurses cannot use professional knowledge properly [31]. In fact, it is a reasonable expectation that in an environment, which is consistent with organized standard of care, basic ethical working conditions are met.

Although patient care is important for nurses, deficiency of clinical standards negatively affect nurses’ performance [32]. This study showed that the effects of environmental factors including facilities and equipment on professional ethics have not been widely reported in the literature [31]. This indicates that deficiencies in clinical settings, such as lack of efficient organization, control and supervision are acutely felt in Iran. Based on the participants’ perspective, another important aspect in compliance of professional ethics is the existence of human resources. Bennett et al. [33] reported that both time and staff shortage and/or in some cases the presence of too many patients are major barriers that challenge nurses in using research evidence and observance of professional ethics in health care. Merakou et al. [34] stated that nurses have in close contact with patients and have a good situation to support them; however, such a role is ignored in Greek hospitals due to staff shortage, lack of enough time and proper training regarding these subjects. Participants In a study conducted by Borhani et al. [32] mentioned that excessive work and staff shortage are two important factors that reduce the quality of care and ethical issues. They also stressed that even if the nurses wish to do so, it is not possible to provide adequate ethical nursing care [35].

The fourth main categories in this study are support systems. Studies showed that elements of supportive environment in nursing contain an appropriate team work, accepting sense of personal identity, freedom to ask questions, and having a suitable working relationship. These factors can enhance professionalism and autonomy in nursing. From practical point of view, however, most nurses have not experienced such a supportive working environment; too much effort is needed to get support [36]. In other studies, inappropriate feedback and insufficient support from both managers and organizations were mentioned by the participants as factors that decrease ethical sensitivity [37]. In this regard, participants in the study of Borhani et al. [32] reported that inadequate support systems were major causes of moral sensibility reduction. Participants of this study believed that when a person was sensitive to an issue, receiving support from others could compensate the inabilities and deficiencies and empower this sensitivity, while inadequate support could suppress this sensitivity [35]. Weaver and Mors [27] stated that inadequate support shared among the managers and colleagues can cause decreased job satisfaction resulting in decreased ethical sensitivity and increased moral distress.

The fifth main category of this study was educational and cultural development. Experts have explained that establishing bonds of commitment to nursing profession depends on cultural considerations [24, 38]. This, in turn, will lead to the enhancement of professional ethics in clinical practices. In doing so, the need for cultural understanding and establishing effective relationships with patients is widely expected to be inserted in the curriculums designed for nursing. Another external factor influencing professional ethics as reported by participants of this study was their desire for an efficient educational system. Nurses, as significant agents of human resources in health care services, play a major role in health promotion of society. Therefore, training programs of nursing should contain materials that incorporate boarder needs of society. Also such programs should be modified according to the changes and advancements in the medical care [39]. Teachers who have theoretical and professional knowledge in the field of ethics can be considered as role models; in fact, they could assist the development of professional ethics [32]. Woods [40] emphasized that although the role of instructors as role models in creation of student’s ethical behavior is important, student’s philosophical readiness and knowledge development in ethical field are the responsibilities of nursing instructors. A wide range of studies are emphasized the effects education on increasing compliance andethical sensitivity. In a conducted review study by Borhani et al. [41] were mentioned that education and training methods could effect on ethical sensitivity. Grundstein-Amado [42] reported that doctors and nurses were not able to properly make an ethical decision and follow a consistent pattern, mainly due to their lack of education in ethical issues. In addition, Wehrwein [43] believed that ethics education improves student’s awareness from ethical issues and their application in the workplace is effective. Moreover, students attending ethics courses were more able in decision making for ethical issues compare to those who did not attend such courses [43]. Rodmell [44] suggests that curriculum is an effective factor in shaping peoples’ attitude and increasing their knowledge, and also a framework to discuss and criticize the ethical issues. Furthermore, he claims that ethical knowledge is an important issue in nursing. In fact, including ethical issues in the curriculum is an appropriate way to be assured of increased ability in solving the ethical dilemmas as well as improved ethical judgment [44].

The research findings have shown that both internal and external factors affect professional ethics in clinical practice. Therefore, professional ethics is not limited to the internal factors. External factors including instructors, administrators, health care providers, education, and culture can be applied in workplace in order to assist nurses in moral development.

## Conclusions

The acquisition of professional ethics is facilitated by internal and external factors. These factors could lead to legitimate norms and standards govern professional behavior of nurses in their relationships with patients. Furthermore, good communication among health care members, improvement of organizational preconditions, appropriate supportive system, and development of education and culture could lead to observing professional ethics in clinical practice.
